# Patients' Educational Program Could Improve Azathioprine Adherence in Crohn's Disease Maintenance Therapy

**DOI:** 10.1155/2020/6848293

**Published:** 2020-04-20

**Authors:** Lei Wang, Rong Fan, Chen Zhang, Liwen Hong, Tianyu Zhang, Zhengting Wang, Jie Zhong

**Affiliations:** Department of Gastroenterology, Ruijin Hospital, Shanghai Jiaotong University School of Medicine, Shanghai, China

## Abstract

**Aim:**

To determine the risk factors of nonadherence to azathioprine (AZA) maintenance therapy for Crohn's disease (CD) and to evaluate the influence of patients' educational program on adherence to AZA maintenance therapy.

**Methods:**

Patients receiving AZA as maintenance therapy for CD were enrolled. Demographic data, clinical data, and usage information were collected. Univariate and multivariate analyses were performed to identify the risk factors of nonadherence. Then, patients' educational program was conducted. One year after the program, the improvements in adherence and relapse rates were compared between educational and noneducational groups.

**Results:**

A total of 378 CD patients receiving AZA as maintenance therapy were enrolled from September 2008 to September 2018. Nonadherence occurred in 43.9% (166/378) of patients. Univariate analysis revealed that young age, education, alcoholism, anxiety, depression, concern belief, and lack of necessity belief and AZA knowledge were risk factors of nonadherence (*P* < 0.05). Multivariate logistic regression showed that anxiety (OR 6.244, 95% CI 2.563–15.213), depression (OR 3.801, 95% CI 1.281–11.278), and concern belief (OR 19.531, 95% CI 3.393–120.732) were independent risk factors of nonadherence. Necessity belief (OR 0.961, 95% CI 0.925–0.999) and AZA knowledge (OR 0.823, 95% CI 0.758–0.903) were protective factors of adherence. One year after the AZA educational program, the necessity belief, AZA knowledge, and adherence of the educational group significantly improved (*P* < 0.05). Concern belief was significantly lower in the educational group than that in the noneducational group (*P* < 0.05). Moreover, the noneducational group suffered significantly higher endoscopic relapse rates than that the educational group (15.9% vs. 30.1%, *P* = 0.035).

**Conclusions:**

Nonadherence occurred frequently in CD patients receiving AZA maintenance therapy. Educational programs could improve patients' adherence mainly by promoting their beliefs and knowledge of AZA and could reduce relapse rates during treatment.

## 1. Introduction

Crohn's disease (CD) is a chronic inflammatory disease that is characterized by periods of remission and relapse and has an unpredictable course [1]. CD treatment includes two different phases: induction and maintenance of remission [2]. For most CD patients, the duration of maintenance therapy far outweighs that of induction therapy. Unlike Western countries, immunosuppressants, such as azathioprine (AZA), remain a mainstay in long-term maintenance therapy in developing countries, including China, where biological agents are not covered by medical insurance in most areas [3–5].

Medication nonadherence occurs in 30%–45% of patients with inflammatory bowel disease (IBD) [6, 7] and is associated with disease exacerbation, poor clinical outcomes, and increased economic burden [8, 9]. Improving adherence during maintenance therapy is of great importance in long-term CD management [10]. However, nonadherence to AZA maintenance therapy for CD has not been fully explored, especially in China. Further identifying the risk factors of nonadherence and investigating potential ways to improve adherence may be considerably important in CD maintenance therapy. In this study, we analyzed the medication adherence of CD patients to AZA maintenance therapy and determined the influence of educational programs on adherence and disease outcomes.

## 2. Methods

### 2.1. Patient Enrollment

The institutional ethics board approved our study. Informed consent was acquired from all patients. This was a single-center cross-sectional and observational study. CD patients receiving AZA maintenance therapy for at least 6 months from September 2008 to September 2018 were enrolled. The diagnosis of CD was based on morphological (radiological, endoscopic, or surgical findings) and pathological criteria suggesting focal, asymmetrical, transmural, or granulomatous features [11]. Each patient experienced remission with Crohn′s Disease Activity Index (CDAI) < 150 through induction therapy with either corticosteroids or biologic agents. AZA doses were adjusted in accordance with side effects and blood tests in a stepwise manner to reach the maximal tolerated dose (1.0–1.5 mg/kg/day) [12]. Patients receiving other concomitant treatment drugs, such as methotrexate, 5-aminosalicylic acid, and corticosteroids, were excluded.

### 2.2. Assessment of AZA Adherence

AZA adherence was evaluated by using the Medication Adherence Report Scale (MARS), a four-item version of the questionnaire used in IBD [13]. Each question has a five-point scale and produces a score between 4 and 20. Similar to other studies, this work defined adherence as MARS 17 to 20.

### 2.3. Assessment of AZA Medication Beliefs

Beliefs about AZA medication were evaluated by using the Beliefs about Medicines Questionnaire (BMQs) [14]. The BMQs included two parts with five-point Likert scales: the belief of medication necessity and concerns about potential adverse effects. Each part involved five-item questionnaires with scores ranging from 5 to 25. Higher scores indicated greater belief of medication necessity or concerns about potential adverse effects. Belief of necessity and concerns about potential adverse effects were separately calculated, with scores of 15 to 25 defined as high medication beliefs or concerns. Medication acceptance was defined as having high necessity and low concern scores, which may be correlated with improved medication adherence.

### 2.4. Assessment of AZA Knowledge

AZA knowledge was evaluated with a 10-item questionnaire designed specifically for Chinese patients (see Supplement). The questionnaire provided 1 point for each question. Total scores ranged from 0 to 10 with higher scores indicating better AZA knowledge. This questionnaire included treatment indication, dose, cessation, side effects, surveillance, and pregnancy and was believed to reflect the understanding and knowledge of AZA.

### 2.5. Analysis of Nonadherence to AZA Therapy

Demographic data, including gender, age, marriage, offspring, education, family income, disease cost, smoking, and alcoholism, were collected and compared to determine the risk factors for nonadherence. Clinical characteristics included disease duration, age of onset, disease location, disease behavior, perianal disease, CD-related surgery, anxiety, and depression. AZA usage information, such as AZA dosage, duration, necessity beliefs, concern beliefs, AZA knowledge, and side effects, was also recorded. Univariate and multivariate analyses were performed to determine the risk factors of nonadherence.

### 2.6. AZA Education and Its Impact on CD Maintenance Therapy

During October 2018 to September 2019, we conducted an AZA educational program for patients. This program was carried out by physicians of our department and consisted of face-to-face classes, on-line classes, and WeChat push. The main contents of this program focused on medical treatment and surveillance of CD under AZA treatment. Nonadherent patients were advised to participate in this program. After 1 year, we compared the psychological status, dosage, medication beliefs, AZA knowledge, adherence, side effects, and relapse rates between educational and noneducational groups. Surgical relapse meant that patients underwent bowel surgery due to CD-related complications. Clinical relapse was defined as CDAI > 150, and endoscopic relapse was regarded as Simple Endoscopic Score for Crohn's Disease exceeding 2 compared with the baseline [15]. We thus determined the impact of AZA education on CD maintenance therapy.

### 2.7. Statistical Analysis

Statistical analysis was performed using SPSS 22.0 (SPSS, Inc., Chicago, IL). Continuous variables are displayed as means ± standard deviations and compared using Student's *t* test, Mann–Whitney test, or one-way analysis of variance. Categorical variables were compared using Fisher's exact test or Chi-square test. Multivariate analysis was performed with logistic regression to identify factors associated with nonadherence using covariates found to be significant by univariate analysis. Statistical significance was regarded as *P* < 0.05.

## 3. Results

### 3.1. Univariate Analysis of the Risk Factors of Nonadherence to AZA Maintenance Therapy for CD

A total of 378 CD patients who received AZA as maintenance therapy from September 2008 to September 2018 were enrolled in our study. Nonadherence occurred in 43.9% (166/378) of patients. Univariate analysis revealed that among demographic parameters, young age, high educational level, and alcoholism were risk factors of nonadherence (*P* < 0.05) (Table 1). Gender, marriage status, offspring, family income, disease costs, and smoking did not significantly differ between the adherence and nonadherence groups. Moreover, the nonadherence group had significantly higher levels of anxiety and depression than the adherence group (*P* < 0.05) (Table 1). Other clinical parameters, such as disease duration, age of onset, disease location, disease behavior, perianal disease, and CD-related surgery, did not significantly differ between the two groups. Furthermore, we found that patients' low necessity belief and AZA knowledge and high concern belief were risk factors of nonadherence to AZA usage (*P* < 0.05) (Table 1). AZA duration and side effects were not associated with patients' adherence.

### 3.2. Multivariate Logistic Regression on the Risk Factors of Nonadherence to AZA Maintenance Therapy for CD

We conducted multivariate logistic regression for the parameters that had univariate significance. We found that on the one hand, anxiety (OR 6.244, 95% CI 2.563–15.213), depression (OR 3.801, 95% CI 1.281–11.278), and concern belief (OR 19.531, 95% CI 3.393–120.732) were independent risk factors of nonadherence. On the other hand, necessity belief (OR 0.961, 95% CI 0.925–0.999) and AZA knowledge (OR 0.823, 95% CI 0.758–0.903) were protective factors of adherence (Table 2).

### 3.3. Effects of AZA Education on AZA Usage in Nonadherent Patients

**Figure 1:**
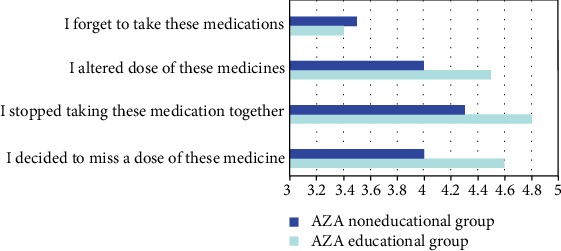
The impact of AZA educational program on different aspects of MARS.

As shown above, AZA belief and knowledge had a great impact on nonadherence. Thus, we carried out an AZA educational program to determine if it would be beneficial to improve adherence among patients. This program enrolled 38.0% (63/166) of nonadherent patients. After 1 year of follow-up, the levels of necessity belief, knowledge, and adherence of the educational group were significantly higher than those of the noneducational group (*P* < 0.05). By contrast, the concern belief of the educational group was significantly lower than that of the noneducational group (*P* < 0.05) (Table 3). For different dimensions of adherence in MARS, we found that “altered dose,” “stopped medication,” and “intentional miss” improved under AZA education, whereas “forgot medication” was similar between the two groups (Figure 1). Other parameters, such as anxiety, depression, dosage, and side effect, did not display significant differences between the AZA educational and noneducational groups.

### 3.4. Effects of AZA Education on Disease Relapse in Nonadherent Patients

**Figure 2:**
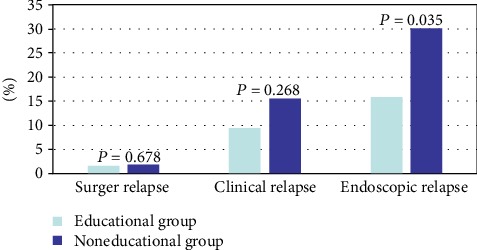
The effects of AZA educational program on disease relapse.

We evaluated the clinical and endoscopic activity of nonadherent patients 1 year after the educational program. During 1 year of follow-up, 1.6% (1/63) patients in the educational group experienced surgical relapse compared with 1.9% (2/103) in the noneducational group (*P* = 0.678). The clinical relapse rate of the AZA educational group was 9.5% (6/63), which was lower than that of the noneducational group (15.5%, 16/103) without significant difference (*P* = 0.268). Moreover, the noneducational group suffered significantly higher endoscopic relapse rates than that the educational group (15.9% vs. 30.1%, *P* = 0.035) (Table 4) (Figure 2).

## 4. Discussion

Patients with CD have a high proportion of nonadherence likely because they are young and have a long quiescent period. Nonadherence to medication in maintenance treatment might directly lead to later flare-ups [8]. Demographic and clinical characteristics have some correlations with medication adherence, but most of them could not be intervening targets. The patients' knowledge and beliefs of medication, which could be influenced by their education, have tight associations with adherence [16]. Moreover, the role of patients' educational programs toward improving adherence remains controversial and has never been studied in CD maintenance therapy with immunosuppressants [17–19]. This study identified the risk factors of medication nonadherence in CD maintenance therapy with AZA and further explored the influence of patients' educational programs on adherence among patients with CD.

The nonadherence rate for maintenance therapy with AZA in our study was 43.9% with a MARS mean value of approximately 16.5, a value that is slightly higher than that reported for patients in Western countries [6, 7, 20]. This result can be attributed to the mild-to-moderate disease courses experienced by patients with CD in China, whereas patients in Western countries are more likely to experience severe activity [21]. Moreover, the patients in our study underwent AZA monotherapy as maintenance therapy. Quiescent disease may lead to the high rate of nonadherence.

We found that nonadherence was associated with the following demographic parameters: young age, high educational level, and alcoholism. This association might be due to the high self-righteousness and low medication necessity belief of young patients and patients with high educational levels. Alcoholism indicates poor self-management ability, which might lead to nonadherence. Moreover, clinical characteristics such as disease duration, age of onset, disease location, disease behavior, and perianal lesions did not have a significant impact on adherence. These findings were similar to those of other research [22–24]. Furthermore, high anxiety and depression levels, low necessity belief and AZA knowledge, and high concern belief were risk factors of nonadherence. These five parameters were further proven to be significant through multivariate analysis. This finding consequently indicated that adherence has more social and psychological instincts than physical-related instincts.

Patients' educational programs might modify medication beliefs and knowledge [25, 26]. However, whether they can improve adherence and disease outcome and their impact on AZA usage in CD maintenance remain to be seen. In our study, we proved that AZA educational programs could significantly improve patients' adherence in three out of four aspects. These improvements might be attributed to the effect of the program on medication beliefs and knowledge. Our results proved that enhancing the patients' belief and AZA knowledge could improve adherence to AZA maintenance therapy for CD.

The AZA educational program may have a positive effect on disease outcome in CD maintenance [27, 28]. This study showed that after 1 year of follow-up, the endoscopic relapse rate of patients in the educational group was significantly lower than that in the noneducational group. Meanwhile, the clinical relapse rate in the educational group was also considerably lower than that in the noneducational group. These findings further proved that AZA education is important in CD maintenance therapy.

Our study has several limitations. First, this is a single-center study and lacks long-term follow-up. Second, although all nonadherent patients were invited to attend our educational program, a few participated in the program. This behavior might prevent us from assessing this intervention comprehensively. Also, we should pay additional attention to the adherence of patients who did not participate in this program to alert possible disease flares. In the future, a multicenter research with larger patient number and longer follow-up should be encouraged to validate the effect of the program.

In conclusion, nonadherence occurred frequently in CD patients undergoing AZA maintenance therapy in China. Anxiety, depression, low necessity belief and knowledge, and high concern belief were independent risk factors of AZA nonadherence. The educational program conducted in this study could improve patients' adherence mainly by changing their beliefs and knowledge of AZA. Consequently, it could also reduce CD relapse rates during treatment.

## Figures and Tables

**Table 1:** Univariate analysis of risk factors for nonadherence in AZA maintenance.

Features	Adherence (*n* = 212)	Nonadherence (*n* = 166)	*P* value
Male sex	130 (61.3)	89 (53.6)	0.132
Age	33.6 ± 9.1	29.7 ± 11.9	0.034
Married	108 (50.9)	71 (42.8)	0.114
Offspring	92 (43.4)	72 (43.4)	0.996
Education			<0.001
Primary school	10 (4.7)	5 (3.0)	
Secondary school	31 (14.6)	10 (6.0)	
High school	73 (34.4)	40 (24.1)	
College	93 (43.9)	90 (54.2)	
Postgraduate	5 (2.4)	21 (12.7)	
Family income per month			0.614
>10 thousand USD	20 (9.4)	17 (10.2)	
5-10 thousand USD	30 (14.2)	21 (12.7)	
2-5 thousand USD	58 (27.4)	49 (29.5)	
1-2 thousand USD	79 (37.3)	52 (31.3)	
<1 thousand USD	25 (11.8)	27 (16.3)	
Cost of disease per year			0.328
>10 thousand USD	51 (24.1)	34 (20.5)	
5-10 thousand USD	81 (38.2)	76 (45.8)	
<5 thousand USD	80 (37.7)	56 (33.7)	
Smoking	8 (3.8)	13 (7.8)	0.087
Alcoholism	4 (1.9)	11 (6.6)	0.019
Disease duration (yrs)	4.5 ± 4.0	4.3 ± 4.0	0.749
Age of onset			0.509
<17 years old (A1)	18 (8.5)	19 (11.4)	
17-40 years old (A2)	177 (83.5)	131 (78.9)	
>40 years old (A3)	17 (8.0)	16 (9.7)	
Location of lesions			0.201
Ileum (L1)	89 (41.9)	85 (51.2)	
Colon (L2)	30 (14.2)	19 (11.4)	
Ileocolon (L3)	93 (43.9)	62 (37.3)	
Behavior			0.422
Nonstricture nonpenetrating	144 (67.9)	102 (61.4)	
Stricture	44 (20.8)	41 (24.7)	
Penetrating	24 (11.3)	23 (13.9)	
Perianal disease	69 (32.5)	49 (29.5)	0.528
CD-related surgery	42 (19.8)	39 (23.5)	0.387
Anxiety	4.2 ± 3.0	7.6 ± 4.0	<0.001
Depression	5.1 ± 3.9	7.5 ± 4.0	<0.001
AZA usage			
Dosage (mg/d)	68.4 ± 35.2	62.3 ± 32.9	0.082
Duration (months)	33.8 ± 24.7	36.1 ± 26.9	0.157
Necessity belief	17.9 ± 2.1	15.4 ± 3.5	<0.001
Concerns belief	14.6 ± 3.0	17.1 ± 2.1	<0.001
Knowledge	6.3 ± 3.1	4.4 ± 2.7	0.028
Side effect	28 (13.2)	19 (11.4)	0.606

**Table 2:** Multivariate analysis of risk factors for nonadherence in AZA maintenance.

Variables	Odds ratio	95% CI	*P* value
Anxiety	6.244	2.563-15.213	<0.001
Depression	3.801	1.281-11.278	0.016
Necessity belief	0.961	0.925-0.999	0.045
Concerns belief	19.531	3.393-120.732	0.003
Knowledge	0.823	0.758-0.903	0.038

**Table 3:** The effects of AZA education on anxiety, depression, and its usage in nonadherence patients.

Features	AZA educational group (*n* = 63)	AZA non-educational group (*n* = 103)	*P* value
Anxiety	7.1 ± 3.2	7.5 ± 4.1	0.723
Depression	7.2 ± 3.1	7.3 ± 4.3	0.674
AZA usage			
Dosage (mg/d)	71.6 ± 34.9	70.2 ± 35.8	0.741
Necessity belief	17.3 ± 2.7	16.2 ± 4.1	0.012
Concern belief	16.3 ± 3.2	17.3 ± 2.6	0.041
Knowledge	7.0 ± 2.9	4.7 ± 2.7	<0.001
Adherence	17.3 ± 2.0	15.8 ± 3.0	<0.001
Side effect	8 (12.7)	15 (14.6)	0.736

**Table 4:** The effects of AZA education on disease relapse in nonadherence patients.

Efficacy evaluation	AZA educational group (*n* = 63)	AZA noneducational group (*n* = 103)	*P* value
Surgery relapse	1 (1.6)	2 (1.9)	0.678
Clinical relapse	6 (9.5)	16 (15.5)	0.268
Endoscopic relapse	10 (15.9)	y (30.1)	0.035

## Data Availability

All of the data in this manuscript are patients' clinical information, characteristics and several questionnaires that are collected by our researchers. These data are available by contacting the corresponding author through email once the article had been published.

## Supplementary Materials

Supplementary Materials It is the AZA knowledge questionnaire used to measure the level of patient's knowledge to AZA which has been described in the “Method” part of our manuscript.
