# Supplementary material for: Patients' Educational Program Could Improve Azathioprine Adherence in Crohn's Disease Maintenance Therapy
Source: Gastroenterol Res Pract. 2020 Apr 20;2020:6848293. doi: 10.1155/2020/6848293 (PMC7189306; doi:10.1155/2020/6848293)
Supplement: Supplementary Materials — It is the AZA knowledge questionnaire used to measure the level of patient's knowledge to AZA which has been described in the “Method” part of our manuscript. [file 6848293.f1.doc]

**AZA Knowledge Report Scale (AKRS)**

1. Which type of medication does AZA belong to?
2. Biologics B. Immumo-suppressants C. 5-aminosalicylic acid D. Corticosteroids
3. What is the advantage of AZA and IFX combined therapy?
4. Higher efficacy B. Reduce the level of IFX antibody C. Both of the above D. None of the above
5. In which phase is AZA more likely to be recommended in CD treatment?
6. Induction of remission B. Maintenance of remission C. Disease relapse D. Any phase
7. When is the best time to end the AZA maintenance treatment if things go all right?
8. 0.5-1 year B. 1-3 years C. 3-5 years D. Not until informed by the physician
9. What is the ideal dosage of AZA in the treatment?
10. Minimal effective dose B. Certain dose for individual C. Maximum tolerated dose D. Not known
11. Which of the following is the most common adverse effect during AZA treatment?

A. Myelosuppression B. Liver damage C. Alopecia D. Pancreatitis

1. What is the routine surveillance test during AZA maintenance treatment?

A. Blood routine test B. Liver function test C. C-reactive protein D. All of the above

1. Which of the following is the target level of white blood cell during AZA treatment? (*10^9/L)
2. Around 3 B. Around 4 C. Around 5 D. Around 7
3. Which of the following is the strongest indicator to stop the AZA treatment?

A.ALT 60U/L B. Fever and cough for 2 days C. Pain and exudation of anal fistula D. WBC 1.0*10^9/L

1. Which of the following should be recommended for AZA treatment during pregnancy?
2. Definitely safe B. Safe, but not as safe as IFX (indicated by clinically validated evidence) C. maybe detrimental D. Definitely detrimental
